# Impact of lossy compression of X-ray projections onto reconstructed tomographic slices

**DOI:** 10.1107/S1600577520007353

**Published:** 2020-07-28

**Authors:** Federica Marone, Jakob Vogel, Marco Stampanoni

**Affiliations:** a Swiss Light Source, Paul Scherrer Institut, Villigen, Switzerland; b Institute for Biomedical Engineering, University and ETH Zurich, Zurich, Switzerland

**Keywords:** lossy compression, X-ray tomographic imaging

## Abstract

Modern detectors used at synchrotron tomographic microscopy beamlines typically have sensors with more than 4–5 mega-pixels and are capable of acquiring 100–1000 frames per second at full frame. As a consequence, a data rate of a few TB per day can easily be exceeded, reaching peaks of a few tens of TB per day for time-resolved tomographic experiments. This data needs to be post-processed, analysed, stored and possibly transferred, imposing a significant burden onto the IT infrastructure. Compression of tomographic data, as routinely done for diffraction experiments, is therefore highly desirable. This study considers a set of representative datasets and investigates the effect of lossy compression of the original X-ray projections onto the final tomographic reconstructions. It demonstrates that a compression factor of at least three to four times does not generally impact the reconstruction quality. Potentially, compression with this factor could therefore be used in a transparent way to the user community, for instance, prior to data archiving. Higher factors (six to eight times) can be achieved for tomographic volumes with a high signal-to-noise ratio as it is the case for phase-retrieved datasets. Although a relationship between the dataset signal-to-noise ratio and a safe compression factor exists, this is not simple and, even considering additional dataset characteristics such as image entropy and high-frequency content variation, the automatic optimization of the compression factor for each single dataset, beyond the conservative factor of three to four, is not straightforward.

## Abstract 2

Synchrotron beamlines acquiring data for tomographic imaging routinely produce a few TB of data per day, and significantly more in the case of time-resolved studies. This work investigates the impact of lossy compression of raw acquisitions onto tomographic reconstructions, aiming for production use. It is shown that a compression factor of at least three to four times does generally not affect the reconstruction quality and that higher factors (six to eight times) can be achieved for tomographic volumes with a high signal-to-noise ratio as it is the case for phase-retrieved datasets.

## 1. Introduction   

Modern sCMOS detectors routinely used at synchrotron imaging beamlines throughout the world feature sensors with more than 5 mega-pixels. With frame rates as high as 100 Hz and the high brilliance and flux of synchrotron sources, full tomographic datasets can be acquired in a few minutes. Advanced CMOS technology, providing frame rates in the kHz regime, pushes the time resolution into the sub-second regime.

The different datasets acquired at Paul Scherrer Institute’s (PSI) TOMCAT beamline (Stampanoni *et al.*, 2006 ▸) can roughly be subdivided into two large classes. Standard datasets consist of 1500–2000 images of about 4–5 mega-pixels each, quantized at 16 bits per sample. A single standard dataset thus amounts to a total of about 15–20 GB, and can be reconstructed to a volumetric image of about 8–15 giga-voxels. Single ‘fast’ datasets, on the other hand, are smaller, since only a fraction of the projections is typically acquired, and show a considerably worse signal-to-noise ratio. In time-resolved experiments, usually, a sequence of multiple ‘fast’ datasets is acquired in a short time span, leading quickly to large data volumes, despite the reduced size of single tomograms.

Raw images are currently not compressed, leading to significant network traffic during data transfer from the detector through the entire tomographic reconstruction pipeline and to storage, both locally and remotely to the users’ institutions. For instance, TOMCAT’s users and staff typically transfer about 1 PB of data to and from PSI’s storage systems every year.

Apart from core operation, the sheer size of tomographic data is problematic with respect to further aspects. The scientific community in Europe and worldwide is moving towards an Open Data Policy: after an embargo period, the acquired data should become available to the community. Scientific journals and national funding agencies (Hahnel, 2015 ▸) are also increasingly requiring open access to scientific data. In this context, large-scale facilities are starting to adopt Open Data Policies and offer long-term archiving options (ESRF, 2015 ▸). The chosen archiving solution might be local on-site, dislocated at super-computing centres or similar specialized institutions, or distributed using cloud technology. Furthermore, efficient data analysis and quantification of such ‘big data’ demands hardware infrastructure not always available on-site: transfer of huge chunks of data to super-computing centres around the planet is slowly becoming routine. Even though cost for TB and transfer rates improve over time, for both storage and transfer, at least in the short term, less data would be extremely beneficial, lead to reduced cost but also immediately significantly ease the every day work thanks to the increased mobility of data.

For these reasons, it is highly relevant to tackle image compression of X-ray projections for tomographic imaging, our ‘raw’ data, and to investigate the effects of doing so onto reconstruction results. The remainder of this section describes related prior work. Section 2 deals with the software infrastructure used for studying the question of how to practically apply and evaluate compression. Sections 3 and 4 present our compression experiments and their results. The remaining sections discuss the outcome and give our conclusions.

### 1.1. Lossless compression   

In terms of image compression methods, one needs to largely distinguish between lossless and lossy methods. Focusing on the former first, the core idea of lossless compression is to recode the original signal in a way minimizing redundancy (maximizing information entropy), thus keeping full information while consuming minimal memory. Well known examples include *gzip* (https://www.gnu.org/software/gzip/) or *bzip2* (http://bzip.org/).

Lossless compression would clearly be ideal, but unfortunately a compression ratio of 1:2 will likely not be exceeded for images typically acquired at third-generation tomographic microscopy beamlines. The lower byte of a typical 16-bit X-ray projection acquired with ‘standard’ settings is particularly affected by noise. Noise is, by nature, non-redundant (*i.e.* of high information entropy) and thus hardly compressible. Even if the higher byte was to compress ideally, the lower byte would be kept, leading to the said ratio.

Actual lossless compression experiments of sample datasets with different lossless compression algorithms support this theoretical deduction, and real compression ratios are typically worse than the 1:2 prediction.

### 1.2. Lossy compression   

While one can already expect to see a certain size reduction when applying lossless compression, more memory savings require lossy methods. This approach can actually be considered standard in most imaging settings for both professional users and consumers.

In such an approach, the original signal is – to a limited degree – modified such that it compresses better, while remaining sufficiently similar to the original data. Usually, this is done by transforming the signal into sparsified coefficients of a wavelet, cosine or similar basis. Prominent examples for such compression methods are the JPEG image format (Pennebaker & Mitchell, 1993 ▸), or any modern video codec such as H.264 (ITU, 2017 ▸) or H.265/HEVC (ITU, 2016 ▸). When only removing some small coefficients, the image will practically look unaltered, but, from a certain point on, typical artefacts will appear. These artefacts will often look blocky as codecs usually process, for technical reasons, small square-shaped image regions at a time. These blocks become visible as soon as compression is applied excessively.

Lossy image compression has been a highly relevant topic for many years, particularly due to its widespread use on internet websites in general and social media in particular. Quite regularly, new compression methods are suggested, and studies compare the performance of different codecs. For instance, *Mozilla* has extensively compared alternatives for its internet browser *Firefox* (Mozilla Research, 2013 ▸). In general, the focus usually rests on the problem of delivering optimal visual appearance to a human viewer while not exceeding a certain memory limit.

Also in the medical imaging field, to cope with the tremendously increasing volume of digital images, selected studies have investigated the effects of lossy compression on the quality of medical pictures and their diagnostic potential (Erickson, 2002 ▸; Seeram, 2006 ▸; Flint, 2012 ▸). Although compression ratios of the raw (Bae & Whiting, 2001 ▸) or final images between 1:5 and 1:15 seem adequate to guarantee correct medical diagnosis (Koff *et al.*, 2008 ▸; ESR, 2011 ▸), the results vary between studies and strongly depend on the imaging modality and used validation metrics. Despite important efforts towards the promotion of the use of lossy compression, this option is actually still rarely used in the medical field, possibly also because of liability issues.

In the context of microscopic tomographic imaging as done at synchrotron facilities, high resemblance is not enough: the quantitative measurement needs to be as accurate as possible. In a tomographic experiment, the original projections represent the raw measurement. Since the acquisition process is tailored for each single experiment, the tomographic reconstruction is not as standardized as in medical imaging but represents a significant post-processing step that needs manual tuning and optimization. At large-scale facilities, the original raw projections are therefore the data that needs to be transferred from detector to storage as well as curated and archived in a long-term fashion. However, scientific work is not carried out on these raw images, but on the reconstructed tomographic slices. Consequently, to assess the potential effectiveness of lossy compression, the core question is: how much lossy compression can be applied to X-ray projection images before the compression artefacts detrimentally corrupt the resulting tomographic reconstructions?

A recent study (Mason, 2014 ▸) found JPEG 2000 (Taubman & Marcellin, 2002 ▸) to be a promising codec for the compression of tomographic microscopy data. A more methodic investigation (Vogel, 2017 ▸) extended this earlier work to consider different lossy image file formats, and different ways to include compression at different stages of the data processing pipeline. It was shown that a compression factor of about four to five times is realistic. However, these results are based on single slices only, and do not consider full volumetric datasets. Recent work by Mancini *et al.* (2018 ▸), also limited to a few representative datasets and a few slices, confirm these factors, when JPEG XR is used.

## 2. Objective and methods   

In this work, we take a pragmatic approach towards the practical application of lossy compression. We focus on raw projection data, *i.e.* images as acquired prior to flat-field correction and reconstruction, and we investigate the similarity of the final tomograms computed from a complete compressed dataset to the equivalent reconstruction from raw, uncompressed data. To ensure that lossy compression in tomographic microscopy could become common practise in the near future, we believe that this step should be seamlessly integrated in the data acquisition pipeline and happen in an automated way. We aim therefore at a *safe maximal compression factor* that can be enforced by default without significantly altering the qualitative and quantitative information in the tomographic volumes, independently from the type of sample investigated and scientific question. Higher compression factors could be envisaged for selected experiments, but their selection will most probably require human control or support from specifically trained AI algorithms.

### 2.1. Compression   

We compare two well established methods, JPEG 2000 (https://jpeg.org/jpeg2000/index.html) using *OpenJPEG* (http://openjpeg.org/) and JPEG XR (https://jpeg.org/jpegxr/index.html) using *jxrlib* (https://github.com/4creators/jxrlib). Other common image formats such as *WebP* (https://developers.google.com/speed/webp/) or *HEIF* (Hannuksela *et al.*, 2015 ▸) either do not support 16-bit intensity values or, despite their hypothetical suitability, no appropriate implementation seems to exist. They have thus been excluded from further consideration.

In addition to these two standardized codecs, we also consider a makeshift scheme of compressing images using *bzip2* after resetting (suspected) noise bits of low significance, thus removing incompressible signal components (Chen & Chuang, 2010 ▸). Despite its coarse nature, this method has the advantage that it can be easily implemented, even in hardware, and cheaply performed online on large datasets.

In all cases, we control the compression via a target compression factor. Only *OpenJPEG* supports this directly as argument. For the other two methods, we use interval bisection to find the respective parameter – a quality percentage for *jxrlib*, and the number of least-significance bits to be reset for *bzip2* – leading to the desired target factor for a sample subset.

The raw images can be compressed independently. This process can thus be highly parallelized, and its performance depends largely on the number of available concurrent compute nodes and the ability of the storage system to support simultaneous read/write access. As we are using standard formats, optimized hardware components may deliver even higher performance.

The actual implementation used for this study was not optimized for high throughput. It is therefore difficult to discuss in an accurate manner the performance of a production system. We are, however, confident that with appropriate hardware such a system can be designed to provide very fast data compression. In our experience, *jxrlib* tends to be almost twice as fast as *OpenJPEG* and might represent the preferred codec in terms of computational performance.

### 2.2. Evaluation   

In general, quality assessment of tomographic reconstructions is a difficult problem. In our case, however, we are only interested in *additional* errors introduced by lossy compression. Therefore, it is sufficient to treat a reconstruction from uncompressed projections as ground truth, and to evaluate the error of a compressed dataset by comparing it with the former using simple numerical methods.

In this report, we compute the mean structural similarity index measure (MSSIM) (Wang *et al.*, 2004 ▸) with respect to the uncompressed ground truth tomogram, yielding a value between 1 (indicating equality) and 0 (indicating low similarity). We set the constants and exponents of the SSIM formula to the default values suggested by Wang *et al.* (2004 ▸) and follow as well the same strategy concerning the weighting function for computing pixel statistics. We define the dynamic range as the difference between the highest and lowest grey-level value within each full tomographic volume. We restrain instead from any image downsampling recommended for taking the viewing distance into account, since we are interested in changes in the structural information not as perceived by the human eye but more by a machine.

Due to the massive size of the whole reconstructed tomograms, the evaluation of each full 3D volume is done in a layer-wise fashion: for every slice, we compute mean and variance of the SSIM, and store the number of pixels. These individual results are then merged to yield statistics for the entire volume (Chan *et al.*, 1979 ▸; Pébay, 2008 ▸).

Instead of using a general threshold, we define a significant similarity degradation by a drop from a plateau behaviour of the MSSIM value as a function of compression factor and a concomitant increase of the SSIM variance. The appropriateness of this approach has been validated visually.

## 3. Qualitative experiment   

**Figure 1:**
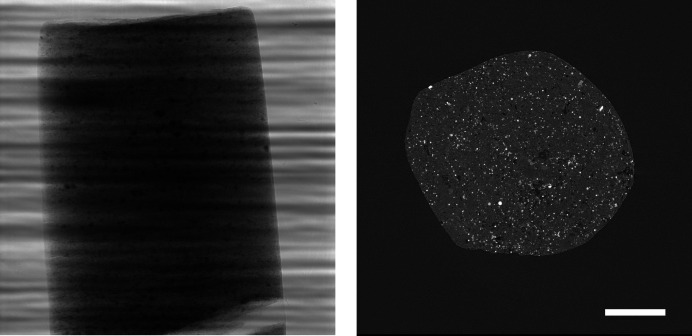
Raw projection image (left, scaled between 6100 and 55000) and tomographic slice (right, scaled between −0.0015 and 0.0045) of dataset *Hornby_b* (Kanitpanyacharoen *et al.*, 2016 ▸). Standard high-quality scan, 1861 projection images of 2048 × 2048 pixels (∼15.6 GB), consisting of 20 dark images (∼167.8 MB), 200 initial flat-field images (∼1.7 GB), 1441 data images (∼12.1 GB), and 200 terminal flat-field images. The pixel size is 0.74 µm and the scale bar corresponds to 300 µm.

**Figure 2:**
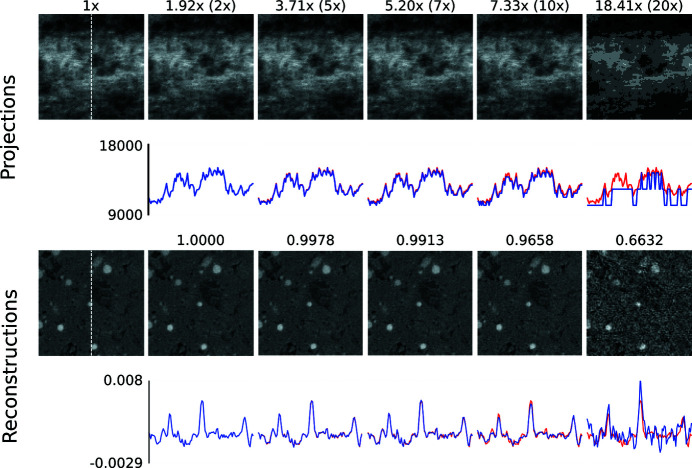
Zoomed (100 × 100 pixels) images of the *Hornby_b* dataset (Kanitpanyacharoen *et al.*, 2016 ▸) after compression using noise bit reset and subsequent lossless compression (*bzip2*). The first column shows the uncompressed ground truth. The top rows show details taken from compressed projection images and respective line profiles (blue is the compressed signal, red the uncompressed ground truth) extracted along the centred vertical axis (indicated by the white dashed line in the left panels). The numbers indicate the actual achieved compression compared with the target one (in brackets). Note the posterization effect in the rightmost image, and the down-rounding of the actual achieved compression caused by the bit reset. The bottom rows show details taken from the corresponding tomographic reconstructions with the respective line profiles. Note the grain effect in the rightmost image. The numbers indicate the computed MSSIM values. The images share the same dynamic range as the line profiles.

In a first step, we present visual results to give an intuition about how compression artefacts affect projections and tomographic reconstructions. We compressed the *Hornby_b* reference dataset (Kanitpanyacharoen *et al.*, 2016 ▸) – a shale sample – using the three different compression methods to a set of different target compression factors. This dataset has been acquired in a ‘standard’ setting: it consists of 1861 projection images – 20 dark-fields, 400 flat-fields and 1441 images with the object – of 15.63 GB in total (Fig. 1 ▸). Figs. 2 ▸, 3 ▸ and 4 ▸ show the resulting images and line profiles for bit reset and subsequent lossless compression, for JPEG 2000, and for JPEG XR, respectively.

Independently of the compression method used, a factor of up to 5× appears to be unproblematic to reach, thus outperforming lossless compression considerably. Even above this factor the results may still be acceptable, depending on the scientific question and the image features the experimenter is interested in.

Differences in the observed artefacts are visible, particularly between the transform-based methods, JPEG 2000 and JPEG XR, on the one hand, and bit reset on the other. For the former two, with increasing compression, projection images begin to show blocks and, within them, blurred ‘wash-out’. These artefacts in the compressed projection images lead to a blurry reconstruction and a loss of contrast.

For bit reset on the other hand, the projections show posterization, *i.e.* originally smooth intensity transitions are turned into piecewise constant plateaus with abrupt changes in-between. Posterization in the projections leads to tomographic reconstructions corrupted by grainy noise. Also note that bit-reset leads to systematic down-rounding, thus biasing reconstructed attenuation values.

## 4. Quantitative experiments   

### 4.1. High-quality datasets   

**Figure 5:**
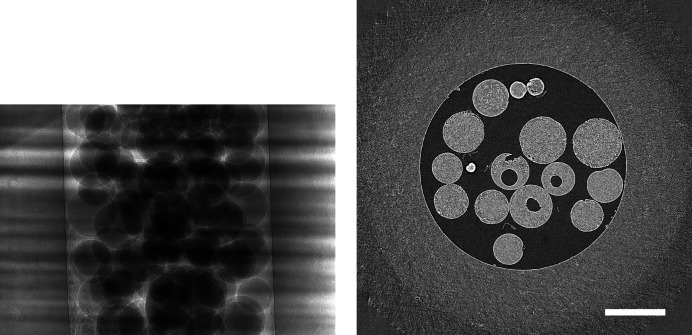
Raw projection image (left, scaled between 3600 and 19000) and tomographic slice (right, scaled between −0.001 and 0.0015) of an Al alloy sample (Marone *et al.*, 2020 ▸). Standard high-quality scan, 1733 projection images of 2560 × 1762 pixels (∼15.6 GB), consisting of 32 dark images (∼288.7 MB), 100 initial flat-field images (∼902.2 MB), 1501 data images (∼13.5 GB), and 100 terminal flat-field images. The pixel size is 0.65 µm and the scale bar corresponds to 300 µm. (Data courtesy of Julie Fife, Paul Scherrer Institut.)

**Figure 6:**
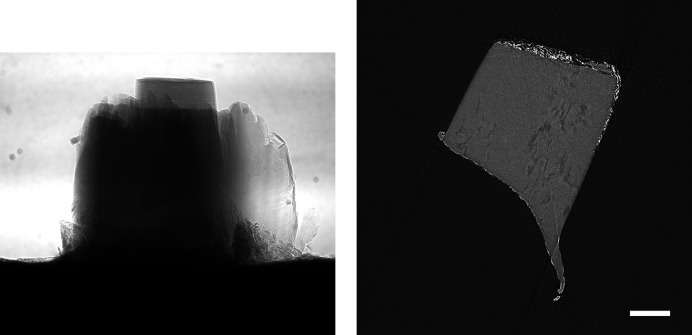
Raw projection image (left, scaled between 100 and 37000) and tomographic slice (right, scaled between −0.001 and 0.004) of a Zr oxide sample (Marone *et al.*, 2020 ▸). Standard high-quality scan, 1711 projection images of 2560 × 2160 pixels (∼18.9 GB), consisting of 10 dark images (∼110.6 MB), 100 initial flat-field images (∼1.1 GB), 1501 data images (∼16.6 GB), and 100 terminal flat-field images. The pixel size is 0.162 µm and the scale bar corresponds to 50 µm. (Sample courtesy of Sousan Abolhassani-Dadras, Paul Scherrer Institut.)

**Figure 7:**
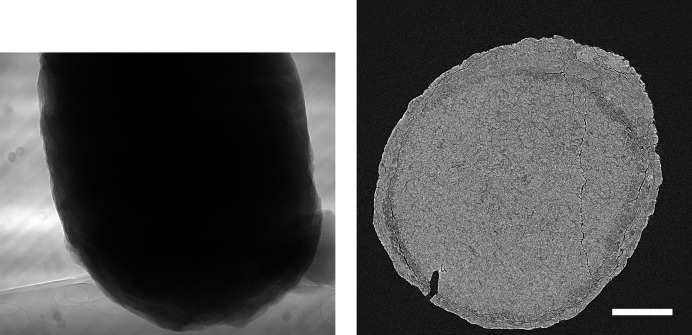
Raw projection image (left, scaled between 8100 and 44000) and tomographic slice (right, scaled between −0.0005 and 0.0011) of a microfossil sample (Marone *et al.*, 2020 ▸). Standard high-quality scan, 1711 projection images of 2560 × 2160 pixels (∼18.9 GB), consisting of 10 dark images (∼110.6 MB), 100 initial flat-field images (∼1.1 GB), 1501 data images (16.6 GB), and 100 terminal flat-field images. The pixel size is 0.325 µm and the scale bar corresponds to 150 µm. (Data courtesy of John Cunningham, University Bristol.)

**Figure 8:**
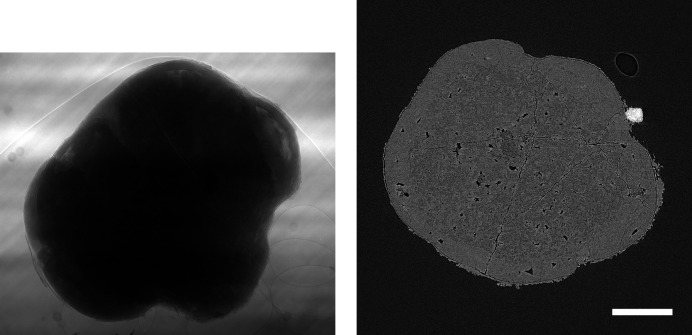
Raw projection image (left, scaled between 10000 and 48000) and tomographic slice (right, scaled between −0.0006 and 0.0018) of a microfossil sample (Marone *et al.*, 2020 ▸). Standard high-quality scan, 1711 projection images of 2560 × 2160 pixels (∼18.9 GB), consisting of 10 dark images (∼110.6 MB), 100 initial flat-field images (∼1.1 GB), 1501 data images (∼16.6 GB), and 100 terminal flat-field images. The pixel size is 0.325 µm and the scale bar corresponds to 150 µm. (Data courtesy of Philip Donoghue, University Bristol.)

**Figure 9:**
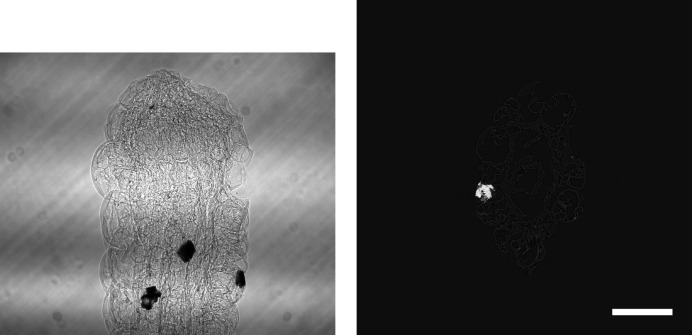
Raw projection image (left, scaled between 14000 and 51000) and tomographic slice (right, scaled between −0.004 and 0.012) of a fossil fruit (Marone *et al.*, 2020 ▸). Standard high-quality scan, 1711 projection images of 2560 × 2160 pixels (∼18.9 GB), consisting of 10 dark images (∼110.6 MB), 100 initial flat-field images (∼1.1 GB), 1501 data images (∼16.6 GB), and 100 terminal flat-field images. The pixel size is 0.325 µm and the scale bar corresponds to 150 µm. (Data courtesy of Else Marie Friis, Swedish Museum of Natural History, Stockholm.)

In order to see whether the behaviour observed for the *Hornby_b* reference dataset generalizes, we have repeated these compression experiments for five further ‘standard’ high-quality datasets with different characteristics (Marone *et al.*, 2020 ▸). Figs. 5–9 show sample projections and tomographic slices for all these datasets. The chosen set of data includes a local tomography scan (Fig. 5 ▸), datasets with small features with low contrast embedded in a homogeneous matrix (Figs. 5 ▸–6 ▸), more complex specimens (Fig. 7 ▸–8 ▸) and edge-enhancement dominated data with the addition of a strongly absorbing particle (high dynamic range, Fig. 9 ▸).

**Figure 10:**
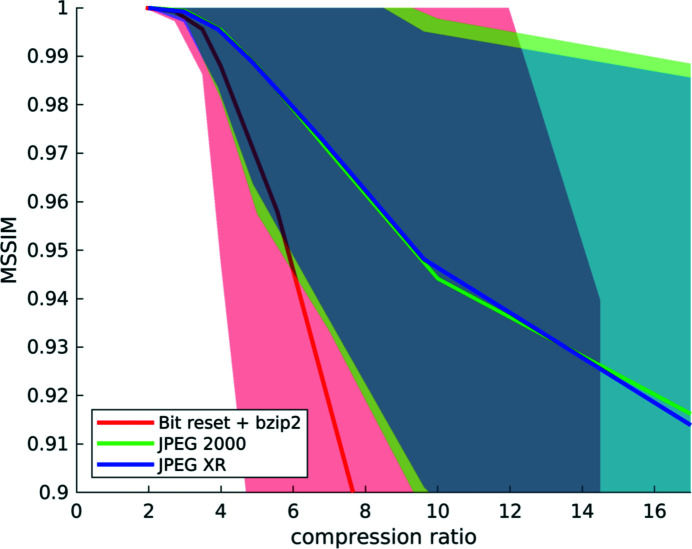
Compression results computed from a set of six different *‘high-quality’* datasets: mean structural similarity and standard deviation over compression ratios for the three different compression methods. Note that a compression ratio of up to about 4× is possible for all three codecs, as the similarity remains high with low variation. After this point, the quality deteriorates quickly for bit reset (red), but also, to a lesser degree, for the two transform-based methods (green and blue).

For every voxel of every dataset, we have computed the structural similarity index, and we have fused all this information to obtain statistics on the behaviour of the three different compression methods with increasing compression factors for *all six* datasets (Figs. 1 ▸, 5 ▸–9 ▸) as explained in Section 2.2. Fig. 10 ▸ shows the obtained similarity curves.

The curves behave largely as described above for *Hornby_b*. The similarity of the reconstructions remains high at low variance up to a factor of about 4×, independently of the compression method. For higher factors, the similarity variance is strongly increasing for all used codecs. The similarity values for the transform-based methods deteriorate instead in a slower manner, compared with those for the reconstructions computed from projections with reset bits. In this latter case the similarity drastically falls for a compression factor higher than 4×. This effect is due to the coarse parameterization of the bit reset compression approach: every additional bit that is reset leads to an exponential loss of information, and compression using this method becomes harder to control with increasing target factors.

### 4.2. Fast measurement   

**Figure 11:**
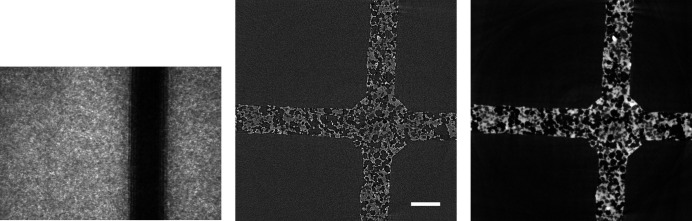
Raw projection image (left, scaled between 250 and 850) and tomographic slices without (centre, scaled between −0.0007 and 0.0012) and with Paganin phase retrieval (right, scaled between 1 × 10^−6^ and 7 × 10^−6^) of a drying porous catalyst structure (Marone *et al.*, 2020 ▸). Fast scan, 1251 projection images of 2016 × 1400 pixels (∼6.75 GB), consisting of 50 dark images (∼270 MB), 100 initial flat-field images (∼540 MB), 1001 data images (∼5.4 GB), and 100 terminal flat-field images. The pixel size is 0.85 µm and the scale bar corresponds to 225 µm. (Data courtesy of Vladimir Novak, Paul Scherrer Institut.)

**Figure 14:**
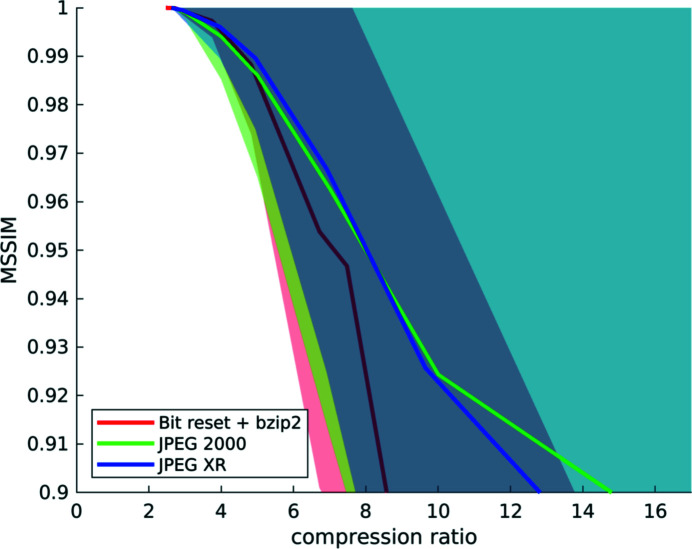
Compression results computed from a set of three different *‘fast’* datasets: mean structural similarity and standard deviation over compression ratio for the three different compression methods. Note that a compression ratio of only up to about 3–4× is appropriate for all three codecs. After this point, the quality deteriorates quickly for all compression methods and the standard deviation increases.

An important feature of the TOMCAT beamline is its ability to image objects volumetrically at high speed. Such a ‘fast’ dataset has typically fewer and smaller projection images with more noise. Consequently, the effect of compression needs to be considered separately for this class of measurements. We investigated three such datasets (Figs. 11 ▸ ▸–13 ▸) (Marone *et al.*, 2020 ▸) in the same way as described above (Fig. 14 ▸).

In comparison with the results obtained for high-quality data (Fig. 10 ▸), a difference in terms of compressibility is visible. ‘Fast’ datasets do not compress as well, and beyond a compression factor of about 3–4× the quality of the reconstructions drops rapidly for all compression methods.

### 4.3. Propagation-based phase contrast   

**Figure 15:**
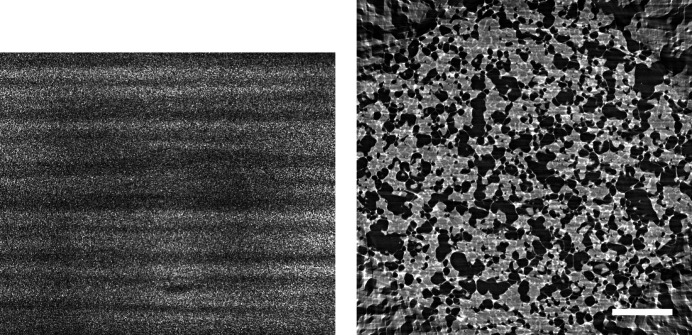
Raw projection image (left, scaled between 400 and 1100) and tomographic slice (right, scaled between −2 × 10^−7^ and 4 × 10^−7^) of an ice cream sample (Marone *et al.*, 2020 ▸). Standard high-quality scan followed by Paganin phase retrieval, 2231 projection images of 2560 × 2160 pixels (∼24.7 GB), consisting of 30 dark images (∼331.8 MB), 200 initial flat-field images (∼2.2 GB), 1801 data images (∼19.9 GB), and 200 terminal flat-field images. The pixel size is 0.65 µm and the scale bar corresponds to 300 µm. (Data courtesy of Annabelle Medebach, Paul Scherrer Institut.)

Many of the datasets acquired at the TOMCAT beamline, and in particular for time-resolved experiments, are not reconstructed directly, but only after phase retrieval, for instance using the Paganin algorithm (Paganin *et al.*, 2002 ▸). In order to see the impact of compression on such an imaging problem, we have considered a further high-quality dataset (Fig. 15 ▸) (Marone *et al.*, 2020 ▸). In addition to that, we have also repeated the compression experiments for the ‘fast’ datasets used in Section 4.2 with phase retrieval.

**Figure 16:**
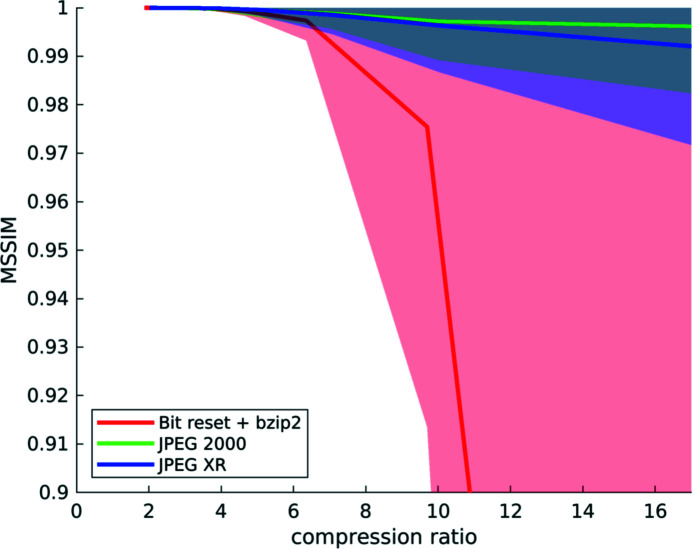
Compression results computed from a single *‘propagation-based phase contrast’* dataset: mean structural similarity and standard deviation over compression ratio for the three different compression methods. Note that a compression ratio of up to about 6–8× seems possible for all three codecs, as the similarity remains high with low variation. After this point, the quality of the bit reset method deteriorates (red) while the other two methods continue to yield good results.

Fig. 16 ▸ shows the similarity curves obtained for the ‘standard’ quality dataset after running the entire reconstruction pipeline using compressed data. This time, the similarity remains high up to a compression factor of about 6–8× for all methods. After this point, the bit reset method begins to show increased variance and the similarity values drop. For the transform-based approaches, the similarity index continues to be relatively high at moderate variance.

**Figure 17:**
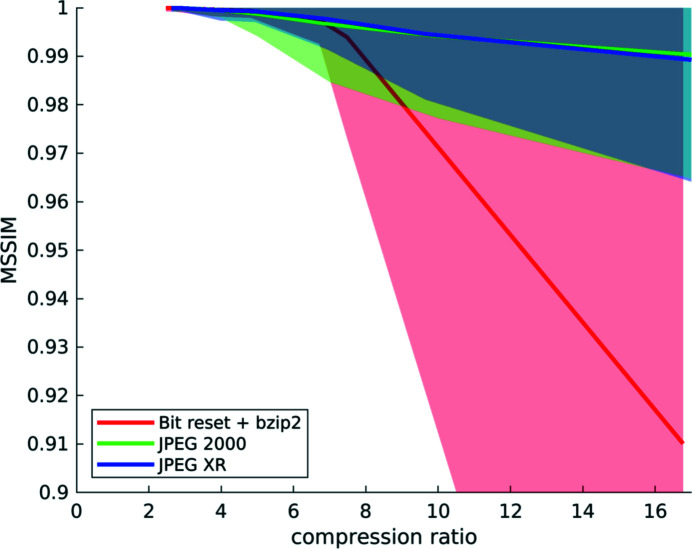
Compression results computed from the three *‘fast’* datasets after retrieval of *‘propagation-based phase contrast’*: mean structural similarity and standard deviation over compression ratio for the three different compression methods. Note that a compression ratio of up to about 6× seems possible for all three codecs, as the similarity remains high with low variation. After this point, the quality of the bit reset method deteriorates (red) while the other two methods continue to yield good results at higher variation.

Fig. 17 ▸ shows the similarity curves obtained for the three fast acquisitions. While following the main pattern seen for the ‘standard’ dataset, although the variance is generally higher, the safe compression factor is about 6× for all methods.

The phase-retrieval algorithm seems to be only marginally sensitive to possible compression artefacts arising in the projections, probably because such artefacts do not typically affect Fresnel fringes but more the smoother parts of the image.

## 5. Discussion   

In this work, we chose to use the mean SSIM as quality metric to quantitatively assess the similarity between the reconstructions obtained from original and compressed projections, as also done in other works on data compression (Mancini *et al.*, 2018 ▸). While image quality metrics are a useful tool to compare results and investigate trends, in our experience they fail in providing absolute references. It was not possible to determine a general MSSIM threshold below which the similarity between datasets started to become significant. Such a threshold was highly dataset dependent. Similar observations were also reported by Mancini *et al.* (2018 ▸). Instead of using a general threshold, we detect a significant similarity degradation by a drop of the MSSIM from a plateau behaviour and an increase of the SSIM variance. The appropriateness of this approach has been validated visually.

To summarize, it appears reasonable to assume that ‘standard’ datasets can be safely compressed by a factor of about 4×, *i.e.* to 25% of their original size. This result indicates that a dataset of 15–20 GB could be turned into one of 4–5 GB, thus promising considerable advantages in terms of storage and network infrastructure.

‘Fast’ datasets allow for less compression, and not exceeding the threshold of about 3–4× appears to be of higher importance. This is likely due to the worse signal-to-noise ratio typically encountered in ‘fast’ settings. Despite their small size of typically ∼4 GB and, therefore, reduced potential with respect to size reduction in absolute terms for a single dataset, tomographic experiments in the ‘fast’ setting modality foresee the acquisition of volumetric datasets in a time-resolved sustained manner with a data rate as high as 8 GB s^−1^ (Mokso *et al.*, 2017 ▸), leading to tens of TB of raw data per day. Despite the need of higher attention in the threshold selection, compression of such datasets is, therefore, of utmost relevance.

Paganin filtering, finally, enables to safely compress the datasets to a higher degree, independently of whether the dataset was acquired in a ‘standard’ or ‘fast’ setting. This behaviour is not surprising, considering the strong smoothing component of the phase retrieval filter that helps to suppress compression artefacts.

**Figure 18:**
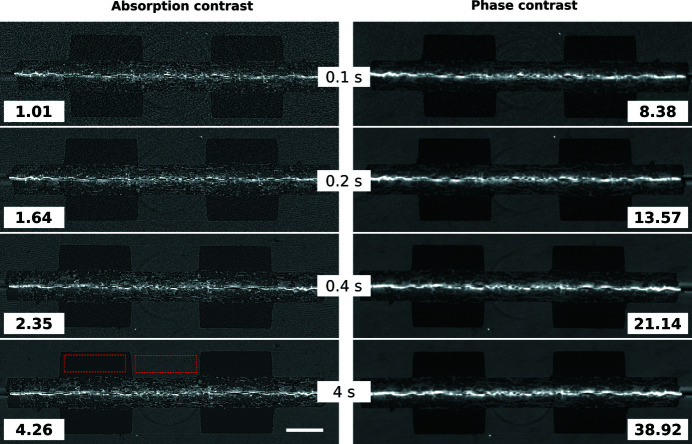
Cropped absorption (left) and phase contrast (right) reconstructed slices through a tomographic dataset of a fuel cell sample (Marone *et al.*, 2020 ▸). From top to bottom the total scan time increased from 0.1 to 4 s. The SNR (calculated using the areas encompassed by the red rectangles) for the full tomographic volumes is indicated by the numbers in the slice corners. The grey-level values for the shown absorption and phase contrast reconstructions are scaled between −0.002 and 0.005, and −3 × 10^−6^ and 1.5 × 10^−5^, respectively. The scale bar corresponds to 400 µm. (Sample courtesy of Jens Eller, Paul Scherrer Institut.)

**Figure 19:**
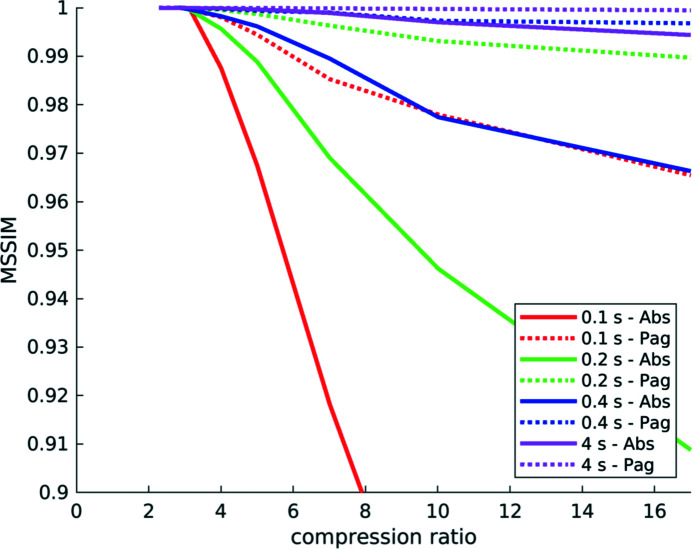
Compression results for the fuel cell datasets shown in Fig. 18 ▸: mean structural similarity over compression ratio for the JPEG 2000 codec. For both absorption (solid) and phase (dotted) contrast dataset suites, a clear trend is observed. With increasing scan time (and SNR) the similarity also increases.

Previous studies (Fidler *et al.*, 2006 ▸) on the relationship between the information of an image and its compressibility indicate that the degree of image degradation strongly depends on image content. Our results suggest also a dependence of the *projection* safe compression factor on the image quality of the *reconstructions*, as previously pointed out by Mancini *et al.* (2018 ▸). The projections of tomographic volumes with low signal-to-noise ratio (SNR) such as those acquired during time-resolved experiments (Figs. 11 ▸ ▸–13 ▸) compress significantly less well than projections of high SNR datasets (Figs. 1 ▸, 5 ▸–9 ▸) acquired with optimally tuned beamline and scan settings. The boost in SNR provided by phase retrieval also contributes to the increase of compressibility of the raw projections. To investigate the dependence of the safe compression factor on the quality of the tomographic volume in more detail, we have repeated the compression experiments for a series of four datasets of the same sample but acquired with a different number of projections and exposure time per projection (Marone *et al.*, 2020 ▸). Cropped absorption and phase-contrast tomographic slices for these datasets are shown in Fig. 18 ▸ together with the average SNR values calculated over the full volume. An increase in SNR of a factor of 4–5× is observed for total scan times going from 0.1 to 4 s. The typical SNR difference between absorption and phase reconstructions is eight- to nine-fold. The compression results for the JPEG 2000 codec are compared in Fig. 19 ▸. For both absorption and phase-contrast dataset suites, a clear trend is observed. With increasing scan time (and SNR), the similarity values also increase confirming the previous qualitative observations. The SNR improvement originating from phase retrieval leads as well to a minimization of compression artefacts up to larger compression ratios as previously discussed (Section 4.3). From this analysis it is though clear that it is not possible to establish a direct relationship between SNR and safe compression ratio. Although for both dataset suites taken individually an increase in SNR leads to a higher compressibility, the 4 s absorption contrast dataset shows higher MSSIM values for most compression ratios than the 0.1 and 0.2 s phase-contrast datasets, despite its lower SNR. The 4 s absorption contrast dataset actually shows similar MSSIM values as the 0.4 s phase contrast dataset, although its SNR is almost a factor of four smaller. These observations lead to the conclusion that, although a relationship between compressibility and reconstruction quality exist, this is not simple and straightforward. The SNR can unfortunately not be used as a unique criterion for the *a priori* blind selection of the optimal compression ratio. The same conclusion is also true if other image characteristics such as the image entropy (Fidler *et al.*, 2006 ▸) or the variation in high-frequency content (Nam *et al.*, 2018 ▸) are considered instead. With these metrics, a similar trend is observed for the fuel cell datasets as the one illustrated for the SNR. Within single datasets it is also possible, at least to some extent, to relate the metric score of each individual reconstructed slice with the visual image impression and for instance distinguish using these metric scores between slices with and without sample. None of these metrics though is successful in capturing and describing the image characteristics in a sample and acquisition setting independent manner. For tomographic volumes of different specimens obtained with different acquisition and post-processing parameters, the relationship between all the investigated metrics and the highest possible compression factor is poor, if not non-existent. These observations suggest that, although in the medical field a combination of image features might be considered for automatically estimating an optimal visually lossless compression factor (Nam *et al.*, 2018 ▸), the same approach fails to address the variability found in synchrotron tomographic data. Based on visual inspection and the limited dataset pool used in this study, the average optimal compression factor is around 8. As a consequence, even if the automatic selection of the highest possible compression factor for each single dataset beyond the safe and conservative ratio of 3–4 would be possible, the additional gain to be obtained with commonly available compression schemes would be ‘only’ of a factor of 2. To significantly push the compressibility of tomographic datasets to factors larger than 100, other avenues, such as video compression (Yan *et al.*, 2019 ▸), might be more adequate.

In the safe compression range, there is practically no difference in compression performance between the results obtained with the different methods. This is an interesting result, considering the technological differences. Even the makeshift scheme of resetting noise bits yields results that are competitive with the more advanced transform-based compression methods. While this approach is easy and fast to implement, caution is needed, though the only input for this method is the number of bits to reset. Considering that there is only a total of 16 bits, and that the significance of individual bits grows exponentially, it is not surprising to see the sudden drop in quality when exceeding the safe threshold. Furthermore, the method likely depends very much on the typical noise characteristics of the imaging setup.

For this reason, we consider one of the transform-based lossy compression methods as the best option for production use, particularly as they would fail graciously in the case of excessive compression: the dataset would be smoother than it could, but not entirely damaged.

## 6. Conclusion and future directions   

We have investigated the impact of lossy compression at the raw projection level onto tomographic reconstructions, and we show that its routine application could lead to a considerable reduction of the produced data volume, potentially resulting in important savings in storage and network infrastructure. In a safe regime, where no deterioration of the reconstructed volume is observed, we expect a general reduction to about 25% of our current resource usage.

This conclusion has been reached using datasets exclusively acquired at TOMCAT. Similar results on the safe compression factor are, however, confirmed by an independent study (Mancini *et al.*, 2018 ▸) using data of the SYRMEP beamline at Elettra. We expect similar results for other facilities as well, since most beamlines share similar setups (*e.g.* similar detectors, microscopes, geometries) and a round-robin study (Kanitpanyacharoen *et al.*, 2013 ▸) confirms that tomographic results are consistent among facilities. For emerging techniques (*e.g.* tomography based on ptychographic projections) and non-parallel geometries (projection and full-field microscopy, for instance) additional investigation is needed, but is beyond the scope of this work.

The three different methods considered in this study show a similar behaviour up to the compression factor of 3–4×, for all datasets investigated. This observation leads us to confidently consider this value as the safe compression factor at least for the datasets acquired at the TOMCAT beamline. Automatic compression of all raw data acquired at the TOMCAT beamline with this established safe factor prior to long-term archiving is currently under consideration and could potentially be performed in a transparent way to the user community.

Higher compression factors might be possible but require additional information on the exact imaging and scientific problem investigated through user interaction (Fritsch & Brennecke, 2011 ▸). A survey of a significantly larger pool of samples might give additional indications in the potential of further reducing the data size.

In this work, we focused on commonly available compression schemes, because these methods are well tested, easily and widely accessible and potentially suited for implementation on hardware. Besides an easier and more efficient implementation at TOMCAT, the choice of such a compression codec would also facilitate inter-operation and data exchange with users’ home institutions, computing centres and other imaging facilities.

These advantages come though at a price. Most importantly, these codecs are based uniquely on intra-frame compression, and the redundancy between neighbouring projection images is not exploited. Such inter-frame compression is quite common for movie codecs, but these might not be directly applicable in our setting. Movie compression exploits the way a human viewer perceives a dynamic scene presented at dozens of frames per second. There is no guarantee that such an approach is appropriate in the tomographic reconstruction setting, even though recent studies show encouraging first results (Yan *et al.*, 2019 ▸). Furthermore, almost no movie codec supports 16-bit intensity values.

Considering that the geometry of tomographic reconstruction is well defined, the motion of image features in projection images could be accurately computed without any assumptions motivated by human perception. Modern movie codecs reach their relatively low data rate by making heavy use of block prediction, and a tomography-specific codec (or tomography-specific block prediction within an existing standard) may be able to optimally compress projection data. Developing and standardizing such a method, however, may be an effort of considerable dimension (beyond the scope of this study), particularly when a hardware implementation is required. Furthermore, coupling the compression of projection images to the versatile process of tomographic reconstruction is far from ideal. Doing so would hinder, or at least strongly limit, *a posteriori* re-evaluation of ‘raw’ data biased by initial choices on the type of reconstruction.

After years of widespread use of JPEG for most purposes despite the availability of more modern formats, recently the field of image compression has been exploring new avenues. One of the leading manufacturers of consumer electronics has extended its operating system to support HEIF (Hannuksela *et al.*, 2015 ▸), a compression format based on the still image component of the HEVC standard (ITU, 2016 ▸). We have considered the same method (wrapped into a different file format) in our earlier work (Vogel, 2017 ▸), but found compression using an available implementation too slow to be realistically applied to our large datasets. Considering though the recently introduced support for consumer electronics, sufficiently fast implementations may soon become available. At that point the potential of HEVC-based image or even movie compression for large tomographic datasets will need to be further assessed.

In addition, the image compression community is also probing entirely new directions, leaving the well known paradigms of coefficient transforms and block prediction. In particular, auto-encoders – first known for pre-training deep neural networks – have recently been investigated for compression purposes (Theis *et al.*, 2017 ▸). Future codecs may thus be able to dynamically adapt to the imaging scenario much more than present-day solutions, and may thus implicitly exploit the predictability of the tomographic problem.

In the meantime, still image codecs look to be a good intermediate solution to significantly safe resources, particularly until dynamic future codecs become widely available. When wrapped into a suitable container format such as HDF5, the compression process may even be transparent, and thus adaptable to future development in the field of compression.

## Figures and Tables

**Figure 3:**
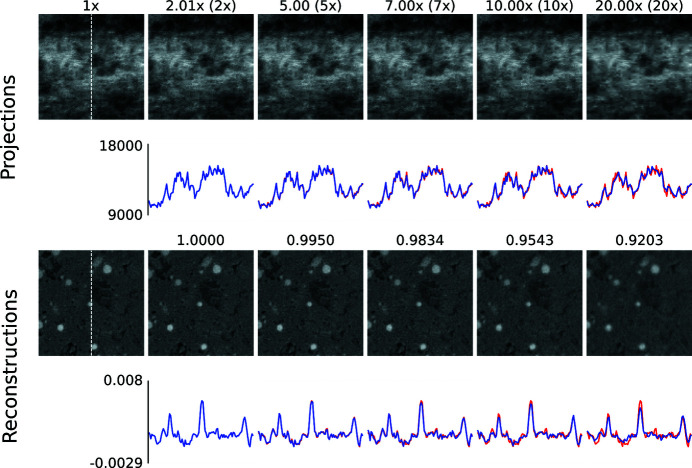
Zoomed (100 × 100 pixels) images of the *Hornby_b* dataset (Kanitpanyacharoen *et al.*, 2016 ▸) after compression using JPEG 2000 (*OpenJPEG*). The first column shows the uncompressed ground truth. The top rows show details taken from compressed projection images and respective line profiles (blue is the compressed signal, red the uncompressed ground truth) extracted along the centred vertical axis (indicated by the white dashed line in the left panels). The numbers indicate the actual achieved compression compared with the target one (in brackets). Note the block artefacts and blur in the rightmost image. The bottom rows show details taken from the corresponding tomographic reconstructions with the respective line profiles. Note the blur in the rightmost image. The numbers indicate the computed MSSIM values. The images share the same dynamic range as the line profiles.

**Figure 4:**
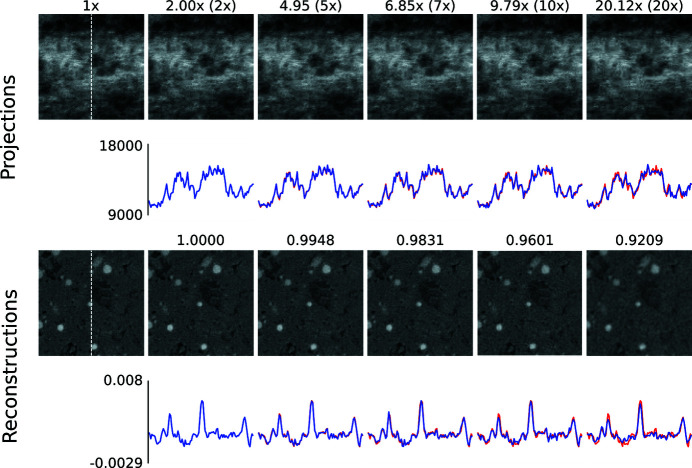
Zoomed (100 × 100 pixels) images of the *Hornby_b* dataset (Kanitpanyacharoen *et al.*, 2016 ▸) after compression using JPEG XR (*jxrlib*). The first column shows the uncompressed ground truth. The top rows show details taken from compressed projection images and respective line profiles (blue is the compressed signal, red the uncompressed ground truth) extracted along the centred vertical axis (indicated by the white dashed line in the left panels). The numbers indicate the actual achieved compression compared with the target one (in brackets). Note the block artefacts and blur in the rightmost image. The bottom rows show details taken from the corresponding tomographic reconstructions with the respective line profiles. Note the blur in the rightmost image. The numbers indicate the computed MSSIM values. The images share the same dynamic range as the line profiles.

**Figure 12:**
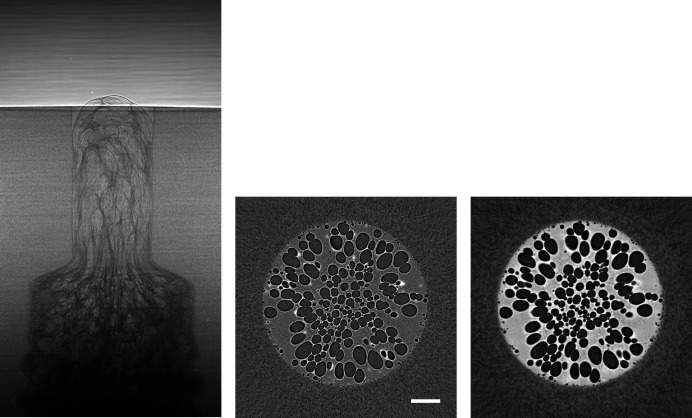
Raw projection image (left, scaled between 400 and 1800) and tomographic slices without (centre, scaled between −0.0025 and 0.0035) and with Paganin phase retrieval (right, scaled between −2 × 10^−5^ and 5 × 10^−5^) of an evolving magma (Marone *et al.*, 2020 ▸). Fast scan, 751 projection images of 1008 × 1900 pixels (∼2.85 GB), consisting of 50 dark images (∼190 MB), 100 initial flat-field images (∼380 MB), 501 data images (∼1.9 GB), and 100 terminal flat-field images. The pixel size is 2.75 µm and the scale bar corresponds to 365 µm. (Data courtesy of Mattia Pistone, University of Georgia.)

**Figure 13:**
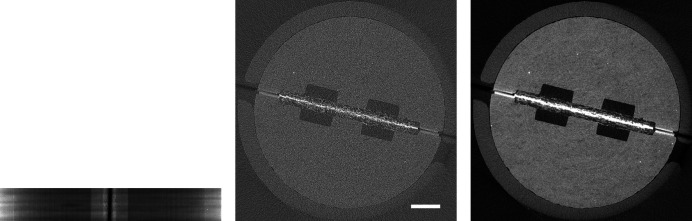
Raw projection image (left, scaled between 70 and 240) and tomographic slices without (centre, scaled between −0.0022 and 0.003) and with Paganin phase retrieval (right, scaled between −1 × 10^−6^ and 6 × 10^−6^) of a fuel cell sample (Marone *et al.*, 2020 ▸). Fast scan, 611 projection images of 2016 × 300 pixels (∼733.2 MB), consisting of 10 dark images (∼12 MB), 100 initial flat-field images (∼120 MB), 401 data images (∼481.2 MB), and 100 terminal flat-field images. The pixel size is 2.75 µm and the scale bar corresponds to 800 µm. (Data courtesy of Jens Eller, Paul Scherrer Institut.)
